# The future of diagnosis in mental health: Promises and challenges of biomarkers to identify reliable and highly predictive biosignatures of affective disorders

**DOI:** 10.1192/j.eurpsy.2025.15

**Published:** 2025-02-06

**Authors:** Alessandra Berry, Mario Luciano, Francesca Cirulli, Andrea Fiorillo

**Affiliations:** 1 Center for Behavioral Sciences and Mental Health, Istituto Superiore di Sanità, Rome, Italy; 2 Department of Psychiatry, University of Campania “Luigi Vanvitelli”, Naples, Italy

**Keywords:** Affective disorders, Biomarkers, Metabolism, Inflammation, Oxidative stress

## Abstract

Current evidence points to a research-practice gap in mental health. There is a specific unmet need to identify novel strategies to improve diagnostic criteria, especially when clinical manifestations overlap as in the case of bipolar (BD) and major depressive disorder (MDD). Based on the rapidly evolving notion that affective disorders are characterized by disrupted brain-body communication, current efforts of neuropsychiatric research are converging towards the identification of specific clusters of peripheral interconnected biomarkers. We argue that these can capture the complexity of the disease as they are linked to the fundamental pathophysiological mechanisms underlying BD or MDD, and can thus deliver an unbiased biosignature. Here we provide a critical viewpoint on the promises and challenges of biomarkers to identify reliable biosignatures of affective disorders. Novel methodological insight and relevant biomarkers are discussed with a main focus on immunometabolic derangements and disrupted redox balance. Major advancements are reviewed taking into consideration that an unbiased diagnosis can only derive from a deep understanding of how biological, psychological, and social factors interact ultimately affecting the clinical manifestation of affective disorders.

Bipolar disorder (BD) and major depressive disorder (MDD) are two of the most prevalent mood disorders, usually associated with significant disability and mortality. Etiologically related, but clinically distinct, they share important clinical overlapping characteristics, which result in higher rates of misdiagnosis. The frequent occurrence of depressive episodes and the later onset of mania in BD subjects often delay a proper diagnosis for years, resulting in greater severity of symptoms, impaired psychosocial functioning, treatment resistance, and higher suicidality. Such delay also leads to a higher number of relapses and hospitalizations, with increased direct and indirect costs associated with the treatment and management of these disorders [1].

The process of diagnosis is quite complex in psychiatry and effective recommendations to improve the differential diagnosis between BD and MDD are lacking. This is of particular relevance, considering that available classification systems are not able to catch the complexity of these mental disorders. In fact, the definition of clear boundaries between psychopathology and the individual variability in behavioral manifestations, as well as between clinical psychiatric entities, is tricky and may result in frequent co-occurrence and heterogeneity within disorders and diagnostic instability over time. This has led to the idea that a categorial approach to mental disorders while representing a milestone in the harmonization of psychiatric diagnoses across different countries, cultures and clinicians, may be too simplistic [2].

Several efforts have been made to overcome the categorical approach to mental illness, one being represented by the Hierarchical Taxonomy of Psychopathology (HiTOP) project, that points to the development of a consensus dimensional classification more clinically informative than the traditional diagnostic systems [3]. HiTOP aims at reducing heterogeneity by defining empirically coherent dimensions and accommodating the widespread comorbidity and poor treatment specificity associated with categorical diagnoses. This is achieved by organizing clinical phenotypes into transdiagnostic spectra and superspectra, which include both psychopathological and neurobiological elements [3].

Another element hindering the achievement of a proper diagnosis relies on the “human factor.” For example, partial or inaccurate psychiatric history reporting and/or the inability to have complete access to previous medical records, as well as the possible inexperience of clinicians, may all represent a source of bias. It is clear that the classical clinical approach alone may be unable to pinpoint objective criteria to formulate a proper and reliable diagnosis, calling for unbiased biological indicators. Such biomarkers and their crosstalk may, therefore, represent the biosignature of the disease, reflecting the fundamental pathophysiologic mechanisms that distinguish MDD from BD [4]. Once identified, such biosignature may guide the diagnosis also offering biological targets for individualized treatment and the identification of novel interventions for BD and MDD.

Affective disorders are characterized by multifactorial etiopathogenesis and complex pathophysiology that result from the interplay between genetic background and epigenetic phenomena occurring during early developmental phases [5]. Moreover, throughout their entire life, individuals will continue to challenge and shape the neural, immune, endocrine, and enteric systems, by actively interacting with the environment ultimately contributing to the heterogeneity characterizing the presentation of clinical symptoms of BD and MDD. Indeed, growing evidence suggests substantial dysregulations of immunological/inflammatory processes and increased oxidative stress (OS) in individuals with affective disorders; these same dysregulations have also been listed in the pathophysiology of metabolic disorders [5].

Not surprisingly, affective disorders are among the psychiatric illnesses most frequently associated with a reduced life expectancy, compared to the general population, mainly as a result of cardiovascular or metabolic comorbidities [5]. Indeed, those experiencing affective disorders with physical comorbidities usually present greater severity of psychiatric symptoms, higher rates of resistance to pharmacological treatments, lower recovery rates, worse long-term outcomes, and reduced psychosocial functioning and quality of life. On the other hand, people suffering from cardiometabolic disorders appear at an increased risk of developing affective disorders throughout life [5]. Taken together, current studies seem to converge on the hypothesis that metabolic and affective disorders may reinforce one another in a vicious cycle relying upon a bidirectional brain – body communication based – at least in part – on immune-inflammatory and OS mediators. Such hypothesis is also strengthened by preclinical research providing evidence for shared common pathways in utero, with prenatal stressors accounting for programmed fetal liability [6]. In fact, it has been recently suggested that, regardless of the specific nature of maternal stress (either metabolic or psychosocial), OS might be one of the main triggers disrupting fetal development [6]. Indeed, the mammalian brain is characterized by poor antioxidant defenses, high metabolic rate, and reduced capacity for cellular regeneration being susceptible to OS and inflammatory insults [7]. Evidence supporting the main role of OS in psychiatric disorders comes from studies on the positive role of antioxidant compounds in a wide range of clinical conditions including MDD and BD. Worth mentioning, specific reactive oxygen species (H_2_O_2_), produced by mitochondria, reinforce insulin signaling promoting metabolic disorders. By contrast, animal models with genetically reduced OS are characterized by altered emotional behavior [7].

From the above-mentioned evidence, it appears clear that changes in the metabolic and redox set-point might play a key role in the pathophysiology and/or in the etiopathogenesis of mood disorders and that immunometabolic pathways may represent a main target in the search for biomarkers. The rationale for searching peripheral signatures of brain disorders relies on the notion, rapidly evolving, that affective disorders are characterized by disrupted brain – body interactions. This ultimately means that a single biomarker, as is the case of some kind of cancers, can hardly represent the specific biosignature of a psychiatric disorder.

Recent efforts to identify disease-specific biomarkers have provided evidence for distinct immune patterns and non-enzymatic antioxidant alterations in BD and MDD. In particular, Gong and co-workers [8] found that levels of circulating non-enzymatic antioxidants might discriminate individuals with BD from those with MDD, while Poletti and colleagues [9], have shown that BD and MDD subjects are characterized by disease-specific immune profiles. Preliminary evidence shows that BD is characterized by an altered metabolic and inflammatory profile during the active phases of the disease [10]. This study is specifically aimed at integrating the immunometabolic and neuroendocrine peripheral biomarkers through a machine-learning approach in the search of a reliable and highly predictive biosignature of the disease. The alterations above described are positively correlated with anxiety, mania, depressive symptoms and the cumulative score for the severity of physical comorbidities. In the same study, when compared to healthy controls, individuals with BD present also a specific immune profile confirming that a selective BD biosignature characterizes the euthymic phase. We are currently extending these findings to include MDD subjects, while refining the biomarkers tested.

At the current level of knowledge, imaging studies represent a valuable non-invasive strategy to complement the study of peripheral biomarkers enhancing the ability to perform a differential diagnosis. However, although promising, this approach is costly and time-consuming, which hampers its massive use in preclinical research. Given the high degree of central and peripheral communication in mood disorders, a promising approach might be to study extracellular vesicles (EV): released from almost all types of cells, including neurons, they may be an additional innovative mean of having direct access to brain-derived molecular markers, and possibly representing a diagnostic implementation of a peripheral, disease-specific biosignature. EV (including exosomes) can cross the blood – brain barrier from both directions and carry proteins, metabolites, and nucleic acids, and once their cargo is delivered into recipient cells, they can alter their immunometabolic response. Although it is still tricky to identify the brain origin of exosomes, this approach is promising in that it provides a “window-into-the-brain” and holds great potential for future advancements in the comprehension of molecular mechanisms underlying psychiatric disorders, their course and outcomes.

Biomarkers can be considered clinically significant when they hold a relevant diagnostic, prognostic, or predictive value. Major advancements in identifying a disease-specific biosignature might derive not only from the characterization of selected biological markers but also from a deep understanding of how they interact with psychological and contextual factors that might affect their expression, ultimately leading to meaningful changes for individuals and the whole health system.

## References

[1] McIntyre RS, Berk M, Brietzke E, Goldstein BI, López-Jaramillo C, Kessing LV, et al. Bipolar disorders. Lancet. 2020;396:1841–56. doi:10.1016/S0140-6736(20)31544-0 33278937

[2] Misiak B, Samochowiec J, Kowalski K, Gaebel W, Bassetti CLA, Chan A, et al. The future of diagnosis in clinical neurosciences: Comparing multiple sclerosis and schizophrenia. Eur Psychiatry. 2023;66:e58. doi:10.1192/j.eurpsy.2023.2432 37476977 PMC10486256

[3] DeYoung CG, Blain SD, Latzman RD, Grazioplene RG, Haltigan JD, Kotov R, et al. The hierarchical taxonomy of psychopathology and the search for neurobiological substrates of mental illness: A systematic review and roadmap for future research. J Psychopathol Clin Sci. 2024;133:697–715. doi:10.1037/abn0000903 39480338 PMC11529694

[4] Abi-Dargham A, Moeller SJ, Ali F, DeLorenzo C, Domschke K, Horga G, Jutla A, Kotov R, Paulus MP, Rubio JM, Sanacora G, Veenstra-VanderWeele J, Krystal JH. Candidate biomarkers in psychiatric disorders: State of the field. World Psychiatry 2023;22:236–62. doi:10.1002/wps.21078 37159365 PMC10168176

[5] Sampogna G, Fiorillo A, Luciano M, Del Vecchio V, Steardo L, Pocai B, et al. A randomized controlled trial on the efficacy of a psychosocial behavioral intervention to improve the lifestyle of patients with severe mental disorders: Study protocol. Front Psych. 2018;9:235. doi:10.3389/fpsyt.2018.00235 PMC6001842 29930520

[6] Musillo C, Berry A, Cirulli F. Prenatal psychological or metabolic stress increases the risk for psychiatric disorders: The “funnel effect” model. Neurosci Biobehav Rev. 2022;136. doi:10.1016/J.NEUBIOREV.2022.104624 35304226

[7] Berry A, Cirulli F. The p66(Shc) gene paves the way for healthspan: Evolutionary and mechanistic perspectives. Neurosci Biobehav Rev. 2013;37:790–802. doi:10.1016/J.NEUBIOREV.2013.03.005 23524280

[8] Gong Y, Lu Z, Kang Z, Feng X, Zhang Y, Sun Y, et al. Peripheral non-enzymatic antioxidants as biomarkers for mood disorders: Evidence from a machine learning prediction model. Front Psych. 2022;13:1–9. doi:10.3389/FPSYT.2022.1019618 PMC9676245 36419979

[9] P Poletti S, Vai B, Mazza MG, Zanardi R, Lorenzi C, Calesella F, et al. A peripheral inflammatory signature discriminates bipolar from unipolar depression: A machine learning approach. Prog Neuro-Psychopharmacol Biol Psychiatry. 2021;105:1–9. doi:10.1016/J.PNPBP.2020.110136 33045321

[10] Di Vincenzo M, Luciano M, Collacchi B, De Felice G, Giona L, Piegari E, et al. The predictive role of proinflammatory and neuroendocrine factors in the acute phases of bipolar disorder: Study protocol of a longitudinal controlled trial. Riv Psichiatr. 2023;58:293–301. doi:10.1708/4143.41409 38032033

